# Dual Functional Magnetic Nanoparticles Conjugated with Carbon Quantum Dots for Hyperthermia and Photodynamic Therapy for Cancer

**DOI:** 10.7150/ntno.91871

**Published:** 2024-04-23

**Authors:** Mounika Choppadandi, Kondi Soumya, Sumanta Ghosh, Aishwarya Balu, Tanvi Shingote, Srivalliputtur Sarath Babu, Vani Sai Prasanna, Somasundaram Arumugam, Ravichandiran Velyutham, Murali M. Yallapu, Govinda Kapusetti

**Affiliations:** 1 National Institute of Pharmaceutical Education and Research, Kolkata, West Bengal, 700054, India; 2 National Institute of Pharmaceutical Education and Research, Ahmedabad, Gujarat, 382355, India.; 3 Department of Immunology and Microbiology, School of Medicine, University of Texas, Rio Grande Valley, McAllen, TX 78504, USA.

**Keywords:** cancer, nanoparticles, magnetic nanoparticles, quantum dots, hyperthermia, photodynamic therapy

## Abstract

The global incidence of cancer continues to rise, posing a significant public health concern. Although numerous cancer therapies exist, each has limitations and complications. The present study explores alternative cancer treatment approaches, combining hyperthermia and photodynamic therapy (PDT). Magnetic nanoparticles (MNPs) and amine-functionalized carbon quantum dots (A-CQDs) were synthesized separately and then covalently conjugated to form a single nanosystem for combinational therapy (M-CQDs). The successful conjugation was confirmed using zeta potential, Fourier transform infrared spectroscopy (FT-IR), and UV-visible spectroscopy. Morphological examination in transmission electron microscopy (TEM) further verified the conjugation of CQDs with MNPs. Energy dispersive X-ray spectroscopy (EDX) revealed that M-CQDs contain approximately 12 weight percentages of carbon. Hyperthermia studies showed that both MNP and M-CQDs maintain a constant therapeutic temperature at lower frequencies (260.84 kHz) with high specific absorption rates (SAR) of 118.11 and 95.04 W/g, respectively. In vitro studies demonstrated that MNPs, A-CQDs, and M-CQDs are non-toxic, and combinational therapy (PDT + hyperthermia) resulted in significantly lower cell viability (~4%) compared to individual therapies. Similar results were obtained with Hoechst and propidium iodide (PI) staining assays. Hence, the combination therapy of PDT and hyperthermia shows promise as a potential alternative to conventional therapies, and it could be further explored in combination with existing conventional treatments.

## Introduction

Cancer is a multifaceted and pervasive disease distinguished by dysregulated cellular proliferation and division, exerting a substantial global impact as a prominent cause of mortality. With an aptitude for local tissue invasion and metastatic dissemination, cancer cells can infiltrate adjacent structures and disseminate to distant anatomical sites. According to the statistical findings reported by the International Agency for Research on Cancer and Cancer Research UK, it is anticipated that in 2022, 1.9 million fresh cancer cases will be diagnosed, accompanied by approximately 609,360 cancer-related fatalities exclusively within the United States. Moreover, the projected global burden of cancer is expected to escalate to an estimated 28.4 million cases by the year 2040, reflecting a 47% rise from the recorded rates in 2020. Furthermore, if current trends persist, cancer incidence in 2070 is forecasted to double compared to the figures observed 1.

Various modalities are currently practiced for cancer treatment, including chemotherapy, surgery, radiation therapy, immunotherapy, targeted therapy, and hormone therapy. However, the treatments are efficient in providing a total cure. Moreover, they have induced more adverse effects and made life more difficult. Hence, there is a vital requirement for alternative treatment to provide a better cure. In this direction, various groups around the globe have been exploring different strategies, primely hyperthermia in conjunction with therapies like radiation therapy or chemotherapy and photodynamic therapy (PDT) for the treatment of tumor(s).

PDT is a type of treatment that uses photosensitizer (PS), when exposed to a specific wavelength of light, it becomes excited and produces a form of oxygen that kills cancer cells 2-4. Porphyrins, chlorins, phthalocyanines, phenothiazinium, squaraine, and other compounds are frequently utilised PS 5. Laser light activates the photosensitizing material, but it has no direct effect on the cells or tissues. In contrast, it gives triplet state energy level to adjacent to oxygen molecules access to it create reactive singlet oxygen (^1^O_2_) species. In the triplet state, it may react in two ways, either by a type-Ⅰ mechanism involving hydrogen atom or electron transfer between the photosensitizer and the target molecule or by a type-II mechanism involving energy transfer to molecular oxygen. Type-I reaction forms free radicals while type-II reaction form singlet oxygen (^1^O_2_). Singlet oxygen causes mainly membrane damage by oxidizing amino acids (tryptophan, cysteine, histidine, methionine, and phenylalanine), unsaturated fatty acids, and cholesterol. Additionally, guanine may also be oxidized by singlet oxygen 6, 7. An ideal photosensitizer can produce the highest reactive oxygen species (ROS) quantity for the PDT effect.

Nanotechnology offers very effective solutions in all dimensions of applications. In recent times, it has predominantly converged toward health care, and quantum dots (QDs) are the prime focus due to their unique physical, chemical, and optical characteristics. Within the QDs family, carbon quantum dots (CQDs) have emerged as prominent contenders for biomedical applications, primarily attributed to their exceptional biocompatibility and minimal toxicity. Moreover, CQDs have garnered recognition as proficient carbon-based photosensitizers, thereby presenting a wide array of opportunities in the realm of PDT. Like its carbon analogs, nitrogen-doped CQDs offer facile modulation of better optical properties. Unfortunately, even the most popular PSs, such as Photolon® and Tookad® bear less fluorescence quantum yield than conventional fluorescent dyes, consequently constraining the theranostic use for simultaneous fluorescence detection and PDT. To address this issue, CQDs were used for upgraded fluorescence detection and PDT 6, 8.

Numerous types of CQDs act as photosensitizers in PDT. The up-conversion nanoparticles (UCNPs) were combined with graphene quantum dots (GQDs) to create a composite that can be employed as a photosensitizer for PDT in the near-infrared therapeutic window. The up-conversion energy transition and dexter excitation transfer were responsible for the ROS-generating performance. The generation of ROS was accompanied by the chemical adsorption of oxygen, which was reduced to O_2_^•-^ by photo-induced electrons and then oxidised to ^1^O_2_ by holes 9. Nitrogen doped CQDs were reported for oxygen photosensitization and singlet-oxygen generation. Further, doxorubicin was loaded into the dots to have a mutual effect of chemotherapy and PDT 10. Sulphur doped carbon dots were claimed for PDT effect in glioblastoma cells by inhibiting PI3K/Akt signalling pathway to treat cancer 11. CQDs conjugated with integrin antagonist arginine-glycine-aspartic acid (RGD) peptides (QDs-RGD) were synthesized and reported for PDT effect in mice bearing pancreatic tumors 12. The increased ROS generation, along with the GQDs favourable biocompatibility and low toxicity *in vitro* and *in vivo* studies, support their potential for use in PDT for cancer treatment 13.

Hyperthermia is a thermal process used to treat cancers by exposing elevated temperatures (42-46 ^o^C) to induce cell death by apoptotic pathway. The treatment employs magnetic nanoparticles with superparamagnetic in nature to produce heat under external alternative magnetic field (AMF) 14, 15. Specific absorption rate (SAR), magnetization, microstructure, particle size, and loss mechanisms are the key factors for heat production 16, 17. Various methods were employed for the synthesis of MNPs includes thermal decomposition, coprecipitation, mechanical dispersion, flame spray synthesis, solvo-thermal, sono-chemical, microwave-assisted, chemical vapor deposition, combustion, laser pyrolysis, and microemulsions 18. Stability of magnetic fluid is one of the critic parameter and it is maintained by decoration of with stabilizers or other functional molecules on top of the MNPs 19. Stabilizers that are commonly used in the synthesis of nanoparticles include polyvinyl alcohol (PVA), polymethacrylic acid (PMAA), polyacrylic acid (PAA), polyethyleneimine (PEI), and citric acid (CA) etc. 20.

Plethora of research was reported on MNPs for magnetic hyperthermia, among superparamagnetic iron oxide nanoparticles (SPIONs) with phospholipid bilayer stabilized nanoparticles studies for magnetic fluid hyperthermia effect in organoids of pancreatic ductal adenocarcinoma to treat cancer 21. Iron oxide MNP with a biocompatible silica shell, conjugated with an antibody against αvβ6 and targeted specifically towards oral squamous cell carcinoma overexpressing αvβ6 and cell death was evaluated following magnetic hyperthermia (MHT) induction 22. MHT was employed to treat peritoneal disseminated lesions using SPIONs coated with carboxydextran that target peritoneally disseminated gastric cancer cells seeded on the omentum in an orthotopic mouse model of gastric cancer 23. Hydrophilic SPIONs *in situ* functionalized with short-chained surface coating molecules (with one or more amine functional groups), such as 1,4-diaminobenzene, 4-aminobenzoic acid, and 3,4-diaminobenzoic acid, and their combination with terephthalic acid, aminoterephthalic acid, trimesic acid, and pyromellitic acid molecules, were reported for treatment of liver (HepG2) cancer 24.

Further, the combination of magnetic hyperthermia and PDT were also explored for effective cancer treatment. In this context, iron oxide nanoparticles (IONPs) coated with novel porphyrin-based photosensitizer drugs were evaluated for their ability to produce singlet oxygen following irradiation for PDT effect 25. Similar fashion, silica coated Fe_3_O_4_ MNPs loaded with curcumin (CUR), a natural photosensitizer, were injected into the tumor site, and tumor was irradiated with continuous wave diode lasers with 450 nm for PDT and 808 nm for photothermal therapy (PTT). The MNPs and curcumin reported effective hyperthermia and singlet oxygen with a PDT effect, respectively 26.

**Figure 1:**
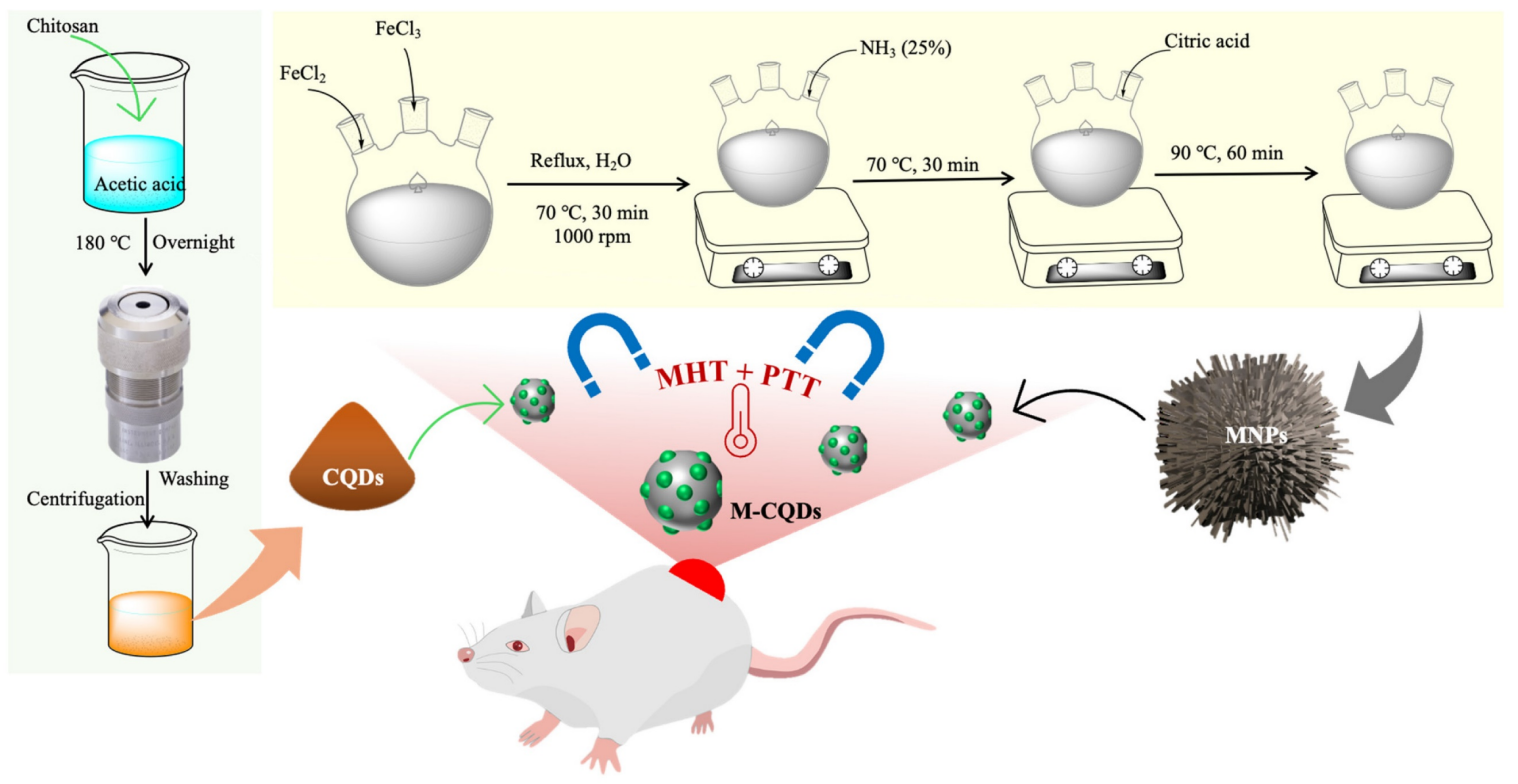
Schematic illustration showing the synthesis procedure of magnetic nanoparticles conjugated with carbon quantum dots (M-CQDs) for dual magnetic hyperthermia and photodynamic therapy for cancer.

In the current investigation, we have synthesized MNPs and A-CQDS separately then fabricated a conjugated system of MNPs and CQDs (M-CQDS) for combinational therapy for cancer (Figure 1). The synthesized nanoparticle has been characterized by using various techniques like FTIR, florescence spectroscopy, TEM, hyperthermia, etc. Further, the M-CQDs are evaluated for *in vitro* hyperthermia and PDT through various cell-based assays.

## 2. Materials and Methods

### 2.1. Materials

Iron (II) chloride (FeCl_2_), ferric chloride (FeCl_3_), chitosan, and 2% acetic acid were purchased from Sigma Aldrich. Ammonium solution 25%, acetylacetone, N-(3-Dimethyl aminopropyl)-N ethyl carbodiimide hydrochloride (EDC), N-hydroxy succinimide (NHS) were obtained from Merck. 3-(4,5-Dimethylthiazol-2-yl)-2,5-diphenyltetrazolium bromide (MTT) reagent, Nitro blue Tetrazolium Chloride (NBT) reagent, Dimethyl sulfoxide (DMSO), F 12-K media were received from Hi-media Laboratories. Citric acid was purchased from SD Fine Chemical Limited. All the chemicals were used without any further purification.

### 2.2. Preparation of Iron oxide nanoparticles (MNPs)

Iron and ferric salts are co-precipitated in a molar concentration of 2:1 (Fe^3+^: Fe^2+^) under non-oxidized conditions to produce iron oxide nanoparticles it gets referred to as "Massart's method 27. Surface functionalization of Fe_3_O_4_ nanoparticles was achieved by using citric acid as a stabilizer. In a typical method, 4.44 g of FeCl_3_ and 1.732g of FeCl_2_ (2:1) were dissolved in 80 mL of Milli Q water in a three necked round bottom flask. The reaction was kept under the refluxing condition at 70 °C temperature for 30 min with constant stirring at 1000 rpm. Then 20 mL of 25% ammonium solution was added to the above reaction and the temperature conditions was maintained for 30 min more. After 4 mL of citric acid solution (0.5 g/mL) was added to the reaction mixture and the temperature gradually increased to 90 °C for 60 min under the same refluxing and stirring conditions. Then the black coloured product was collected from a flask and cooled to room temperature and thoroughly rinsed with water simultaneously the product was separated with the permanent bar magnet (Figure 1).

### 2.3. Synthesis of amine-functionalized carbon quantum dots

The A-CQDs were synthesized by using chitosan as the main precursor in hydrothermal carbonization process (Figure 1). In a typical procedure, 4 g of chitosan was dissolved in 36 mL of 2% acetic acid which was then sealed in a PTFE liner assembled with stainless steel (Teflon liner). This complete setup was placed in a hot air oven/ muffle furnace for hydrothermal treatment at a high temperature i.e., 180 °C for overnight (12 h). After the completion of reaction, the hot air oven was cooled. A dark yellowish-brown solution was obtained which was further centrifuged using centrifuge (Thermo Scientific - Sorvall legend XTR) at 12000 g for 30 min to remove the non-fluorescent or less fluorescent CQDs. The supernatant brown solution exhibited bright green fluorescence under a longer wavelength of 365 nm in the UV chamber 28.

### 2.4. MNP and A-CQD conjugation

The coupling agent (3-Dimethyl aminopropyl)-N-ethylcarbodiimide hydrochloride (EDC) and N-hydroxysuccinimide (NHS) are used for the conjugation. In the experimental procedure, two separate solutions were prepared: one containing 3 mg/mL of N-Hydroxysuccinimide (NHS) added to the A-CQDs and the other containing 4 mg/mL of N-(3-Dimethylaminopropyl)-N'-ethyl carbodiimide hydrochloride (EDC) added to the MNPs. These individual mixtures were then combined in a beaker and subjected to sonication for 30 min. After sonication, the mixture was further treated with a vibrating process using a vortex station for approximately 4 h at a temperature of 5 °C. The concentration of nanoparticles was kept constant and QDs concentration was varied to achieve the different A-CQDs: MNPs ratios respectively. Finally, the product (conjugated MNPs and A-CQDs (M-CQDs)) was collected from the mixture by using a permanent magnet followed by washing with distilled water and then freeze dried for 1 day 29.

### 2.5. Characterization of A-CQDs, MNPs, and M-CQDs

#### 2.5.1. Fluorescence analysis with UV cabinet

For visualization of samples, UV cabinets consist of UV tube lights with longer wavelengths (365 nm), shorter wavelengths (254 nm) and daylight tubes as desired. These UV cabinets are mainly used to analyse the chromatographs by UV fluorescence. Here the UV cabinet was used to examine the fluorescence of the A-CQDs under daylight and longer wavelength with the naked eye (Lalco Scientific Instruments) 30.

#### 2.5.2. Zeta potential

The zeta potential measurements were performed using a Malvern Zetasizer (Nano ZS90 series, UK) based on the dynamic light scattering (DLS) mechanism. A-CQDs, MNPs, and M-CQDs, each at a concentration of 1 mg/mL, were dispersed in Milli-Q water with a pH of 6.8 and conductivity of 0.05 μS/cm at room temperature. The dispersion was sonicated for 15 min and then transferred to a single use zeta cuvette for analysis. The zeta potential was measured by sample size of n=3.

#### 2.5.3. Fourier transform infrared spectroscopy

The Fourier transform infrared spectrometer (FTIR A1, Bruker, Germany) was used to investigate the chemical interactions and functional groups present in A-CQDs, MNPs, and M-CQDs. To perform the analysis, KBr pellets containing approximately 3-5 mg of each sample were prepared using a hydraulic press. The FTIR scans were conducted in the frequency range of 400 to 4000 cm^-1^ with 64 scanning accumulation, comparing the background (plain KBr pellet) and the sample scans. The acquired data were then analyzed using OPUS 7.5 software (Bruker, Germany).

#### 2.5.4. Transmission electron microscopy

Transmission electron microscopy (TEM) (FEI - Tecnai G2, F30) is an imaging help for examining the crystallographic structure and particle size of samples. In this highly energetic electron uses. After allowing electrons to travel through the specimen, an image is created on a fluorescent screen or photographic film detected by the charged couple device (CCD). The synthesized nanoparticles, quantum dots, and conjugated magnetic quantum dots were analyzed by using TEM at 300KV with a point resolution of 0.2 nm the size of the particle.

#### 2.5.5. UV-visible spectrophotometer

The molecular interaction and absorbance maxima were investigated using a UV-Vis spectrophotometer (UV-1800, Shimadzu, Japan). A-CQDs, MNPs, and M-CQDs at a concentration of 0.5 mg/mL were dispersed in Milli-Q water and then sonicated for 15 min. The analysis was conducted in a scan range of 200 nm to 800 nm, using Milli-Q water as a blank for baseline correction.

#### 2.5.6. Heating ability determination of MNPs

The hyperthermia experiments were performed using Magnetometer (Nanotherics). The heating capacity of magnetic nanoparticles and magnetic quantum dots was performed by using a vibrating sample magnetometer / magnetometer (VSM) and a specific absorption rate was calculated. Sample was vibrated by using coil (frequency) and the magnetic field. Upon exposing the optimized concentration of nanoparticles to the magnetic field and radiofrequency, the temperature increase was observed. The specific rate absorption values were given by using the following equation.

SAR = C × ∆T/∆t × 1/ mFe

Where,

SAR = Specific absorption rate,

C = Specific heat capacity of solvent (C _water_ = 4.18 J/g °C),

∆T/∆t = Slope of the time-dependent temperature,

mFe = Mass fraction of iron

#### 2.5.7. Fluorescence spectroscopy

To measures the emission wavelength (spectra) of the quantum dots fluorescence spectrophotometer was used (Horiba - Fluorolog 3-221). The excitation wavelength starts from 320-520 nm with a 20 nm increment with a slit width of 3 nm.

#### 2.5.8. Energy Dispersive spectroscopy

The elemental analysis on the nanocomposite was carried out with an EDS detector (EDAX Ametek, USA) coupled with FE-SEM (Zeiss Gemini - Sigma 300). Samples are coated with gold before the experiments to enhance the sample's conductivity. An accelerating voltage of 5 kV and a scan size of 2 µm were used during the analysis. The elemental analysis was done with an SEM micrograph carrying a magnification of 9299 X and a resolution of 127.4 eV.

### 2.6. Cell viability assay

The degree of cytotoxicity was evaluated for the synthesized MNPs, A-CQDs, and M-CQDs using MTT assay [42]. Briefly, A549 (lung cancer cell line, ATCC) were seeded in 200 μL of culture medium at a density of 10,000 cells/well in a 96-well plate and incubated for 24 h at 37 °C. After 24 h, culture media was aspirated and the cells were treated with 200 μL of media containing different concentrations of the following formulations: 1) Only cells (control), and 2) three variants of MNPs (4, 5, and 6 mg/mL), 3) three variants of A-CQDs (0.5, 1, and 1.5 mg/mL), and 4) three variants of M-CQDs (optimized concentrations are 5mg+0.5, 5mg+1, and 5mg+1.5 mg/mL). Here, 5 mg/mL is MNPs and 0.5, 1, and 1.5 mg/mL are CQDs. After completion of incubation time, cells were further incubated for 3 h with 20 μL of MTT (0.5 mg/mL). After treatment, media was discarded from each well, and subsequently, 200 μL of DMSO was added to solubilize the formazan crystals formed. Readings were taken from a multi-plate reader (Thermo Scientific - Varioskan Lux) at 560 nm using software (SkanIt RE 4.1). Percentage cell viability was calculated using the following equation:



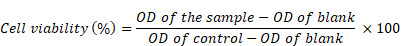



### 2.7. NBT assay

Reactive oxygen species generation (superoxide anions) due to phototherapy is determined by NBT assay. For this study, the A549 cells were grown in a medium at 37 °C in a 5% CO_2_ environment. After the culture has reached 90% confluence, the cells were detached from the culture flask by treatment with a Trypsin-EDTA solution and counted to a cell density of 1×10^4^ by Trypan blue exclusion method and cultured in 96 well plates. After the internalization of quantum dots, cells were exposed to violet light for 30 min and again incubated for 24 h. After that, the 100 μL (6 mg/mL) of NBT reagent was added and incubated for 2 h. After incubation 100μL of KOH and 100 μL of DMSO were added and the optical density was checked at 610 nm 31.

### 2.8. Apoptosis assay

Hoechst staining and PI staining were performed to analyse the morphology of the cells during apoptosis [42]. The nucleus was stained by the Hoechst reagent (blue color) whereas the PI stains the dead cells (red) by intercalating into DNA. For the study 4 x 10^5^ A549 cells were seeded in each polystyrene culture dishes and incubated for 24 h. Then cells were treated with different formulations (MNPs, A-CQDs, and M-CQDs) and incubated for 24 h. After the sample treatment, the culture dishes were washed with 1 mL PBS and stained with Hoechst (10 µg/mL) and PI (15 µg/mL) per dish and incubated for 30 min at 37 °C. Cells were observed under a confocal laser scanning microscope (Leica - LAS SP8 X). The same treats sample culture plates have further proceeded for the nuclear morphology under confocal microscopy 32.

### 2.9. In vivo studies: Assessment of MHT with PDT by M-CQDs

To verify the MHT with PDT effect, we have evaluated the M-CQDs in in -vivo. All animal experiments were performed according to the approval from the institutional animal ethics committee (IAEC) and committee for the control and supervision of experiments on animals (CCSEA). Briefly, 24 female, white Balb/c mice (4-5 weeks' old) were utilized for the study. Initially, MCF-7 cells were cultured for 7 days in T-25 cm2 tissue culture flasks and harvested. Further, the animals were anesthetized with 2% isoflurane and a subcutaneous injection of MCF-7 cells with a volume of 100 μL (1 × 10^7^ cells/ml) were injected near left flank of third breast fat pad of each mouse 43, 44. The mice were kept in a specific pathogen-free facility with a 12-h light/dark cycle and had free access to food and water. The 2-DG (2-Deoxy-D-glucose, PerkinElmer) probe of 10 μL per mouse was given intraperitoneally for detecting solid tumors for the tumor confirmation with IVIS imaging (In vivo Imaging System by PerkinElmer). After 2 weeks, once tumors volume reached approximately 30 mm^3^, the animals were randomly divided into the 4 groups. Group-1 served as the disease control (no treatemnt), while the group-2 received PDT treatment alone with M-CQDs (Fortoo, 12V 1.5W blue LED source with an optic fiber cable). The group-3 was solely subjected to hyperthermia treatment with M-CQDs (Magnetherm, Nanotherics, UK), and the group-4 received combined treatment of PDT with MHT.

Prior to treatment, the animals were anesthetised with 2% isoflurane. Subsequently, they were injected intratumorally with a 100 μl (50 mg/ml) nanoparticle dispersion. Treatment sessions for each group lasted for 30 minutes, with PDT, hyperthermia, and combined PDT with hyperthermia administered at separate intervals on 0, 5^th^, 10^th^, and 15^th^ day. During treatment sessions, the animals' temperatures were monitored using a thermal camera. Furthermore, the animals are imaged in IVIS for the proper and uniform tumor size the animal were subjected for treatment. Based on the intensity of accumulation of the probe in the solid tumor, the region of interest was calculated to measure the tumor volume. Tumor volume was assessed using the IVIS imaging system employing a 2-DG probe at specified time intervals.

#### 2.10. Statistics analysis

Statistical analysis of data has been done by using the software GraphPad prism 5. Statistical tests such as ANOVA have been applied to data to check the significance of the data with Turkey's post Comparison. Here n=3. * Vs Control. *p<0.05, **p<0.01 and ***p< 0.001.

## 3. Results and Discussion

### 3.1. Characterization of A-CQDs, MNPs, and M-CQDs

#### 3.1.1. Fluorescence spectroscopy

**Figure 2:**
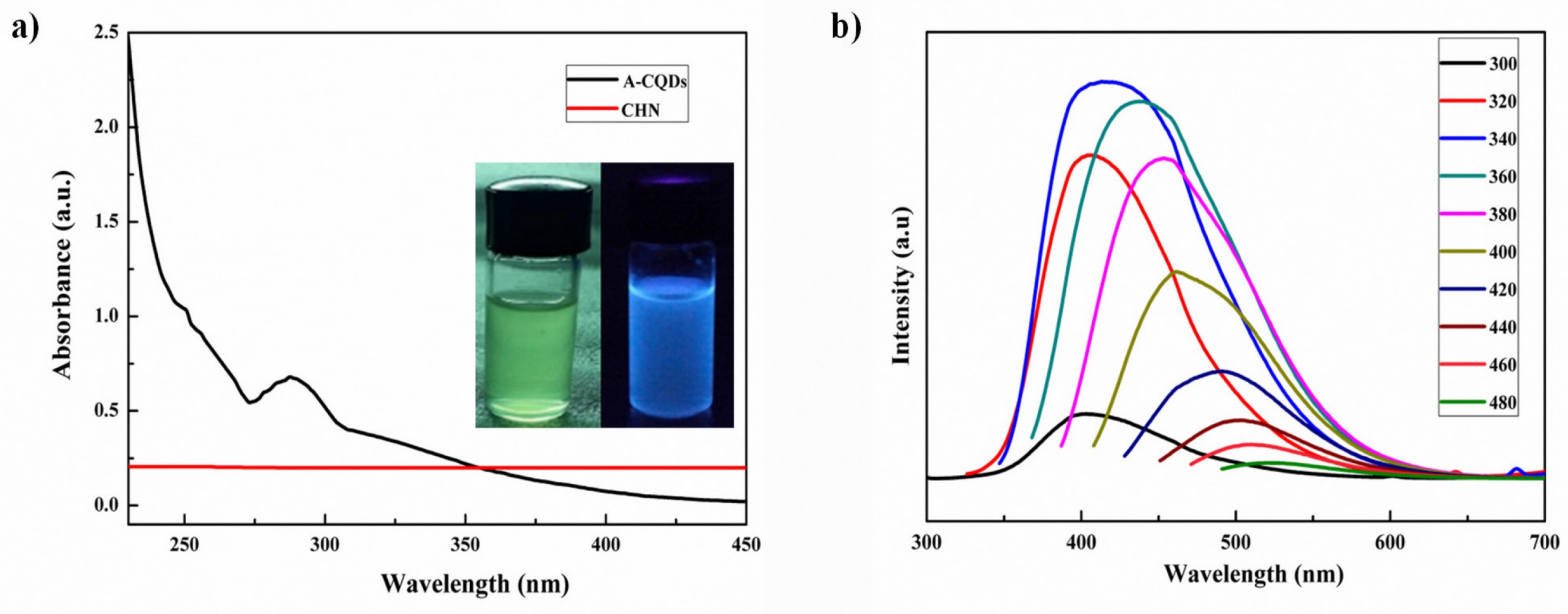
UV-Visible spectra of (a) A-CQDs with λ_max_ of 288, the inserted images represent the florescence (blue color) under UV light exposer, while light yellow bottle is at normal environment and (b) fluorescence excitation-emission spectra of A-CQDs shows the excitation dependent emissions.

A-CQDs were subjected for UV cabinet for visual examination of fluorescence. In the daylight A-CQDs are in transparent yellowish solution and under longer UV light exposer they exhibited strong blue fluorescence. The fluorescence property of quantum dots was preliminarily observed in the UV cabinet and then it was confirmed by UV-visible and fluorescence spectroscopies (Figure 2a). The UV-Visible absorption feature centred at 288 nm and shows absorbance in UV as well as in visible regions. Fluorescence spectrophotometer was used to measure the excitation and emission wavelength (spectra) of QDs. Various excitations (320-520 nm) were given to the A-CQDs with a 20 nm increment with a slit width of 3 nm**.** Excitation dependent emission spectra was observed when subjected to different excitations (Figure 2b). Therefore, the CQDs are demonstrated higher florescence with excitation dependent emissions.

Similarly study for the optical properties of CQDs reported by Liang *et. al*. The group was synthesized CQDs from gelatin and absorption spectra were reported at 250-290 nm with maximum emission at 430 nm for excitation of 350 nm 33. However, the CQDs exhibit limited absorbance in the NIR region to get better penetration and ROS effect. This limitation can be addressed through various approaches such as size modulation of CQDs to influence the electronic band structure, enabling better absorption of red and NIR light 34. Surface modification is another effective method to enhance light absorption properties and extend their range to include the red and NIR regions. Introducing appropriate dopants into the CQD structure can modify their energy levels, leading to improved light absorption in the desired wavelength range. Additionally, creating heterostructures by combining CQDs with other semiconductor materials can further enhance light absorption and broaden the spectral range 34.

#### 3.1.2. Zeta potential

Zeta potential serves as a critical parameter in assessing the stability and interactions of nano systems within formulations. The zeta potential measurements were performed to investigate the surface charge and interactions of A-CQDs, MNPs, and their conjugate, M-CQDs. The zeta potential of A-CQDs was determined to be 11.6 mV, indicating a positive surface charge. This observation strongly suggests successful functionalization of A-CQDs with amine groups, contributing to the positive charge. Conversely, the zeta potential of MNPs was found to be negative (-33.2 mV), indicating the presence of carboxylate (COO¯) groups on the MNPs' surface, likely due to citric acid adsorption.

**Figure 3:**
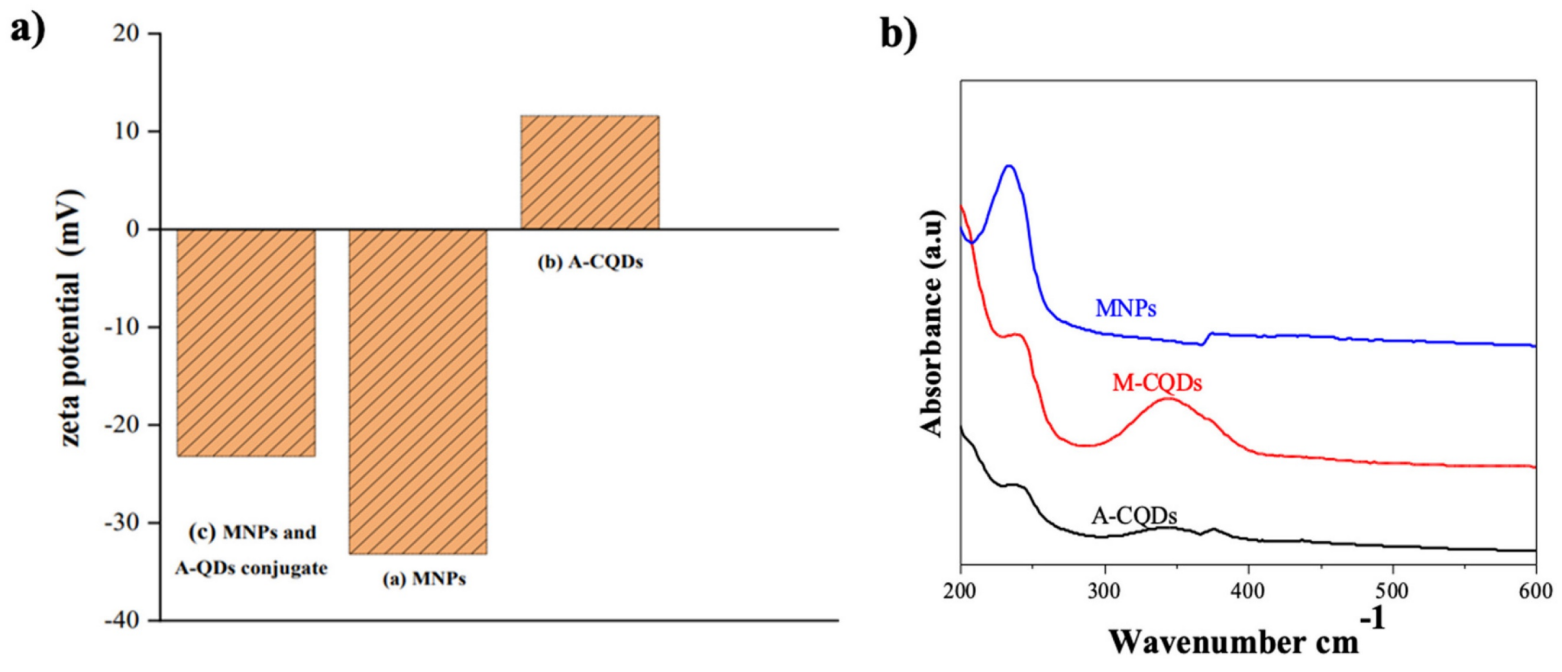
Zeta potential of (a) MNPs (-33.2), (b) A-CQDs (11.6), and (c) M-CQDs QDs conjugate (-23.2), the decreased negative charge in M-CQDs resulted from conjugation of A-CQDs and b) UV-Vis spectra of A-CQDs, MNPs, and M-CQDs.

Upon conjugation of MNPs and A-CQDs, the resulting M-CQDs exhibited a reduced negative charge with a zeta potential of -23.2 mV (Figure 3). The decrease in negative charge can be attributed to the electrostatic attraction between the negatively charged MNPs and the positively charged A-CQDs, leading to a charge transition in the conjugated particles. A similar phenomenon of charge transition was reported in a related study involving up-conversion nanoparticles (UCNPs) and their modifications. The UCNPs and PEGylated UCNPs displayed a positive charge of +30 mV, while graphene oxide-modified UCNPs and hypocretin-A-conjugated UCNPs exhibited negative charges of -8 mV and -3 mV, respectively 35. These findings further support the notion of charge modification occurring during particle conjugation, indicating successful M-CQDs formation in our study.

The UV-visible spectroscopic analysis of A-CQDs, MNPs, and M-CQDs was performed to investigate their optical properties and interactions, as shown in Figure 2b. MNPs exhibited a characteristic absorption peak in the UV region, specifically around 200-300 nm, which is associated with electronic transitions within the iron oxide structure. A-CQDs showed an absorbance peak at 240 nm, attributed to π to π* transitions, as well as another absorption band around 350-400 nm, due to n to π* transitions. The transitions involve the excitation of electrons from non-bonding (n) orbitals to the empty π* anti-bonding orbitals 36. M-CQDs displayed combined features of both MNPs and CQDs, indicating successful conjugation. The UV-visible spectrum of M-CQDs showed transitions with potential shifts of around 5 and 10 nm, respectively, accompanied by clear broadening. Hence, the UV-visible analysis confirming the successful conjugation of CQDs with MNPs.

#### 3.1.3. FT-IR spectroscopy

**Figure 4:**
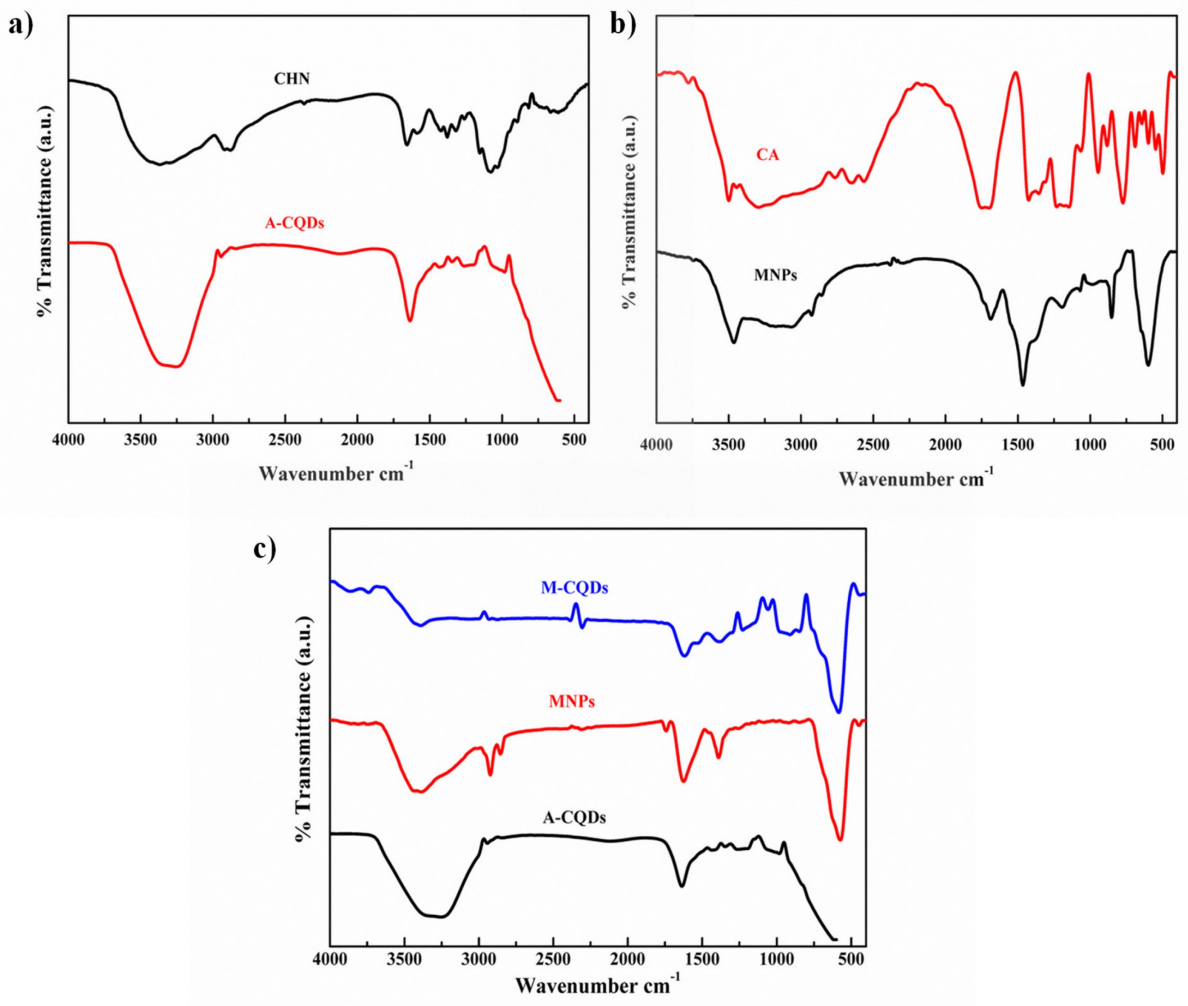
FT-IR spectra of (a) A-CQDs, (b) MNPs, and (c) conjugated QDs and MNPs (M-CQDs).

The functional groups of the synthesized A-CQDs, MNPs, and M-CQDs were analyzed using FT-IR. The pristine chitosan powder exhibited N-H and O-H absorption bands of amine groups at 3448 and 2926 cm^-1^. The C-H stretching was observed at 2850 cm ^-1^. The bands at 1643 and 1592 cm^-1^ indicates N-H bending vibrations. C-H bending vibrations of pyranose ring was observed at 1130- 1064 cm^-1^ 37. The A-CQDs showed decreased adsorption bands of O-H and N-H vibrations at 3438 cm^-1^ compared to pristine chitosan. The C-H vibrations at 1130-1064 cm^-1^ related the pyranose ring were completely dehydrated (Figure 4a) 38. Therefore, it was proved that the A-CQDs were successfully synthesized from the chitosan. FT-IR spectra of MNPs show the peak at 1710 cm^-1^ indicates the C=O asymmetric stretching i.e., the COOH group of CA shifts to 1660 cm^-1^ band of Fe_3_O_4_ nanoparticles revealing the binding of a CA radical onto the surface of Fe_3_O_4_ nanoparticles. The strong band at 585 cm^-1^ band ascribed to Fe-O stretching vibrational mode (Figure 4b) 27. The functional groups of synthesized MNPs were confirmed. The 1660 cm^-1^ (carboxylic group) in MNPs and the amino group in A-CQDs 1643 cm^-1^ together shift towards A shorter wavenumber (cm^-1^) i.e., 1614 cm^-1^ indicating the formation of the amide bond in the M-CQDs due to the EDC-NHS coupling agent (Figure 3c) 29.

#### 3.1.4. Energy dispersive spectroscopy

**Figure 5:**
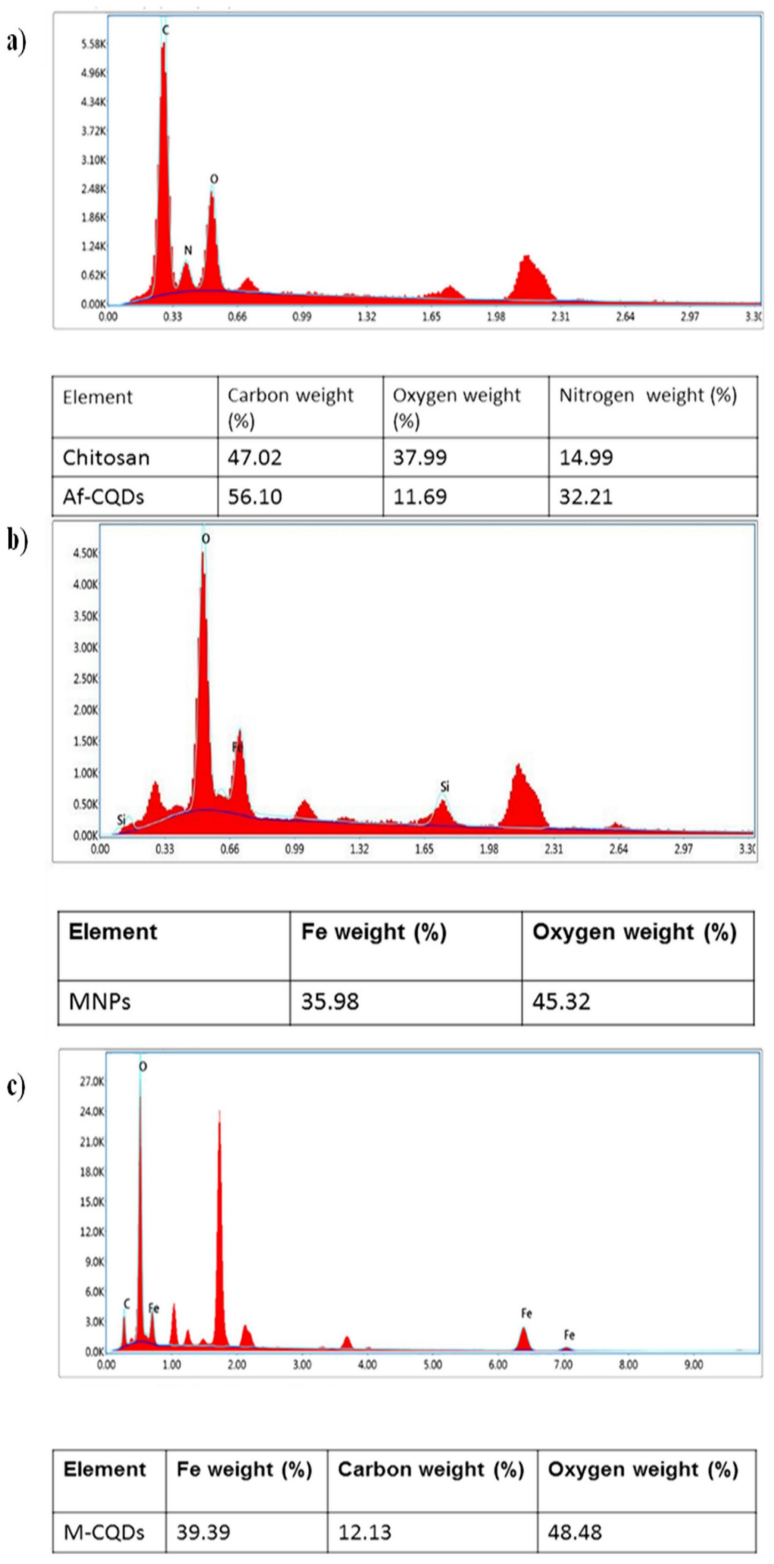
Energy dispersive spectra of (a), A-CQDs (b)MNPs, and (c) M-CQDs.

In the elemental analysis, the percentage of carbon in synthesized A-CQDs was found to be more compared to the neat chitosan indicating that the carbonization (Figure 5a) 28. The elemental composition of synthesized MNPs i.e., iron, oxygen, and carbon was confirmed by EDX analysis (Figure 5b) 27. In conjugated formulation, both the elements i.e., carbon and iron were seen indicating that MNPs and A-CQDs were conjugated. The elemental composition of M-CQDs was shown in (Figure 5c).

#### 3.1.5. Morphology of A-CQDs, MNPs, M-CQDs

**Figure 7:**
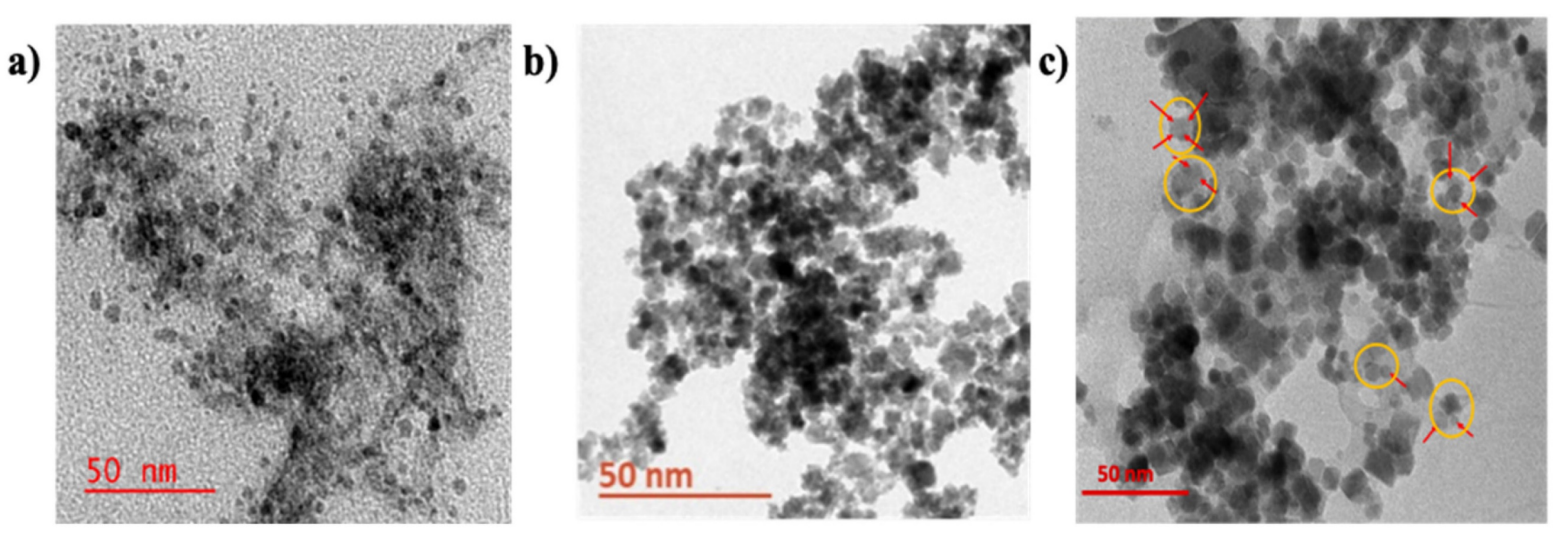
TEM images of (a) A-CQDs of size ~2-8 nm with spherical shape, (b) MNPs with size of 10-20 nm, and (c) M-CQDs of size 20-30 nm, shows conjugated CQDs on top of MNPs the yellow circle represents the M-CQDs, where the red arrows show the CQDs with light grey, while dark black particles are MNPs.

The size and morphology of the A-CQDs, MNPs, M-CQDs were determined using TEM analysis. The A-CQDs show particle size ranging from 2-8 nm with a spherical shape (Figure 7a) (depending on the size of the particles, they exhibit different fluorescence at specific wavelengths, 8 nm size shows red fluorescence, 6 nm for green fluorescence and 2-5 nm for blue fluorescence). Whereas the MNPs show particle size ranging from 10-20 nm (Figure 7b). The M-CQDs showed particle size about 20-30 nm and structure clearly shows the conjugations of A-CQDS on top of MNPs (Figure 7c).

The conjugation of A-CQDs with MNPs via EDC chemistry is an intriguing process that involves considering the citric acid coating on MNPs and the potential impact of NHS conjugation 39, 40. The MNPs were coated with citric acid, which mostly involve one carboxyl group out of three, as shown in the following studies [41] and the other two carboxylic acids remain free, which helps avoid conjugation and stabilization of the MNPs suspension. In the current study, the free carboxylic acid groups were explored for conjugation of A-CQD using carbodiimide chemistry.

### 3.2. Magnetic hyperthermia

The heating capacity of MNPs and M-CQDs were performed by using a hyperthermia system and the specific absorption rate (SAR) was calculated. Different concentrations of samples were taken (4, 5 and 6 mg/ml) and exposed to the fixed magnetic field at different frequencies with a fixed time. The 5 mg/ml concentration showed an increase in temperature with 4 different frequencies. The highest SAR value i.e., 118.11 ± 3.51 W/g was found with the lowest frequency (260.84 kHz) and the same frequency has demonstrated therapeutic temperature (42-46 °C). The heating abilities of MNPs are shown in Figure 7a with variable frequencies. After the conjugation of MNPs with A-CQDs with EDC-NHS the SAR value decreased compared to pristine MNPs. All four pre-sets' frequencies increased the temperature above 42.0 °C but with less SAR values compared to bare MNPs. This is due to the decrease in concentration of MNPs after the conjugation of A-CQDs. M-CQDS with 5 mg/ml exhibited a 95.04 ± 3.16 W/g SAR value but still reached the temperature of 42.0 °C (therapeutic temperature) for long time interval can provide efficient hyperthermia effect for better treatment as compared other concentrations. The heating efficiency of the M-CQDs are shown in (Figure 7b) and SAR values presented in Table 1.

### 3.3. Cell viability assay

**Figure 8:**
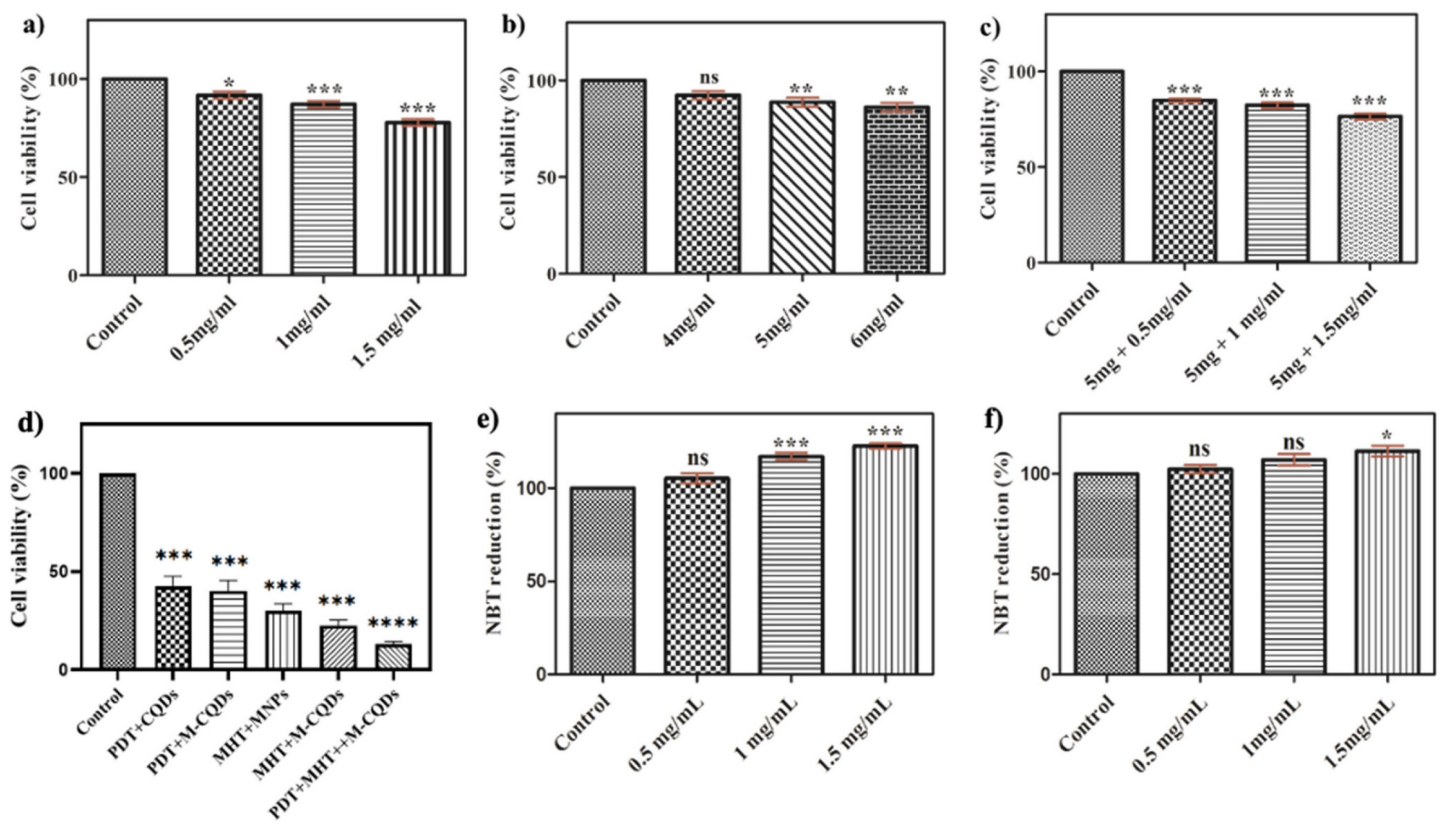
Cell viability analysis of **(a)** A-CQDs, **(b)** MNPs, **(c)** M-CQDs, and **(d)** Cell viability under different therapeutic conditions through MTT assay. Quantitative estimation of reactive oxygen species of **(e)** A-CQDs and **(f)** M-CQDs through NBT assay. All the data expressed as mean ± SEM and analysed for statistical significance using one way ANOVA with Tukey post-test Comparison Test, n=3, * vs Control. **p<0.01, ***p< 0.001.

The A-CQDs, MNPs, and M-CQDs were evaluated for biocompatibility using of A549 cells by incubating them with a medium containing different concentration of CQDs, MNPs, and M-CQDs for 24 h. The cells without formulation were considered as control. Figure 8 shows the percentage viability of cells after 24 h of incubation. The A549 cells were seen to be unaffected with concentrations as high as 1 mg/mL CQDs, 5 mg/mL MNPs, and 5 mg + 1 mg/mL M-CQDs (i.e., MNPs : CQDs = 5 : 1). Hence, A549 cells show tolerance and survival towards all the formulations. Hence, it was determined that the concentration of M-CQDs for hyperthermia was optimized and demonstrated biocompatibility.

Further, the cell viability of A549 cells were determined under various treatment conditions to understand the efficacy of therapy. Significantly, under combinational therapy (hyperthermia +PDT) only 3-5% of cell viability was observed against single hyperthermia (13%) and PDT (38%) suggesting that the combinational therapy is highly effective towards cancer treatment.

### 3.4. NBT assay

Reactive oxygen species (ROS) generation was calculated by using an NBT assay. A549 cells were incubated with different concentrations of A-CQDs, M-CQDs, and control without formulation. After internalization of A-CQDs and M-CQDs, the cells were exposed to a violet laser for 15 min. Further, these cells were incubated for 24 h and 100 μL of 1M KOH, and 100 μL of DMSO were added and optical density was measured at 610 nm. Superoxide anions were generated by xanthine-xanthine oxidase and detected by NBT reduction. NBT is a reagent that can absorb superoxide and change its color to purple. The concentration of CQDs is directly proportional to the generation of ROS (1 and 1.5 mg/mL) while in M-CQDs the generation of ROS hindered due to the conjugation of MNPs (Figure 7. e-f) 31.

### 3.5. Hoechst and PI dyes

**Figure 9:**
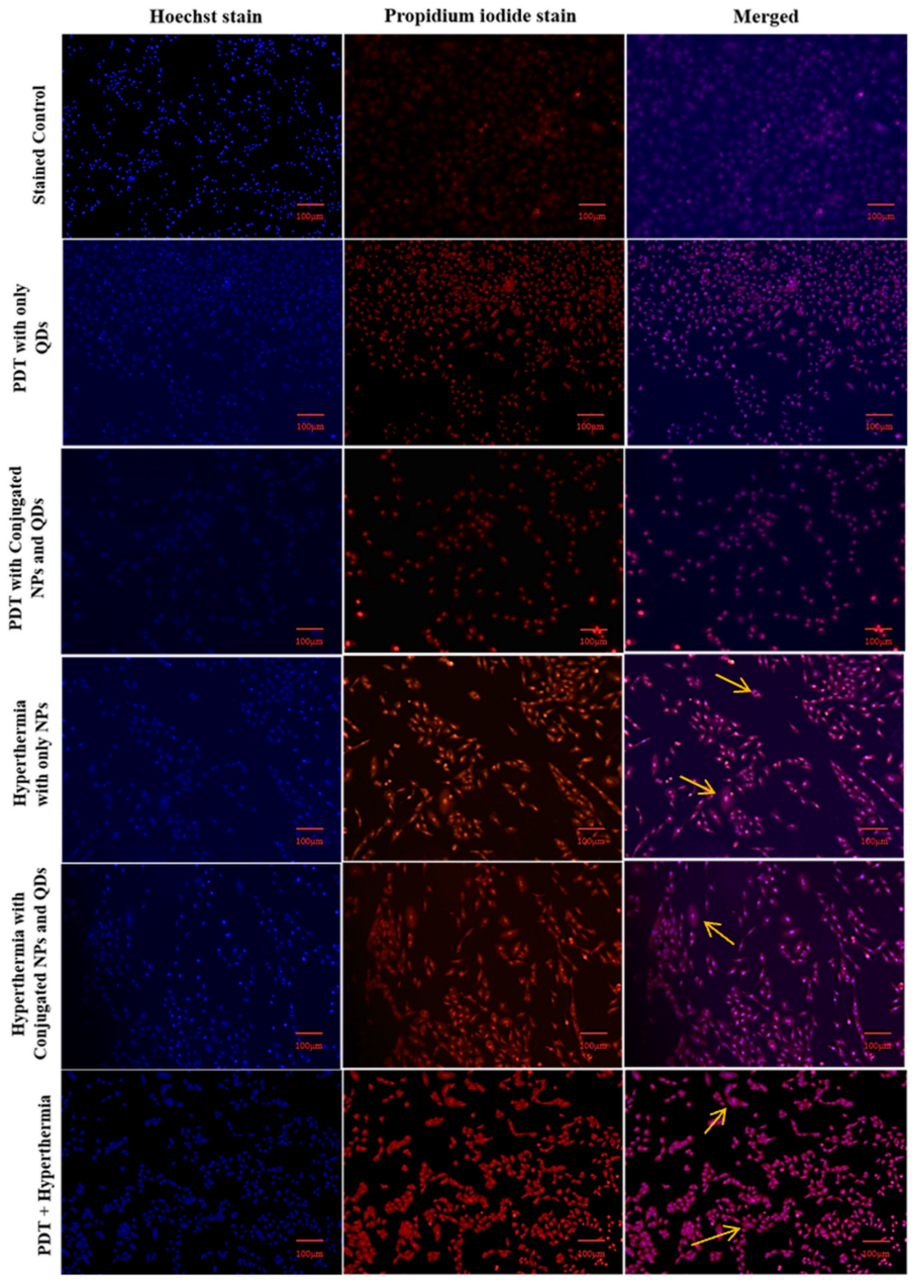
Fluorescence images of A549 cells treated with PDT, hyperthermia, and PDT+ hyperthermia using CLSM (Scale bar: 100 µm).

The staining procedure was carried out to determine the stage of cell death following magnetic hyperthermia and the PDT effect. To distinguish the difference between live and dead cells, Hoechst, and PI stains were used [42]. PI stains the nucleic acids of dead cells with red fluorescence but does not permeate live cells, whereas Hoechst stains the nucleus with blue fluorescence regardless of whether the cells are alive or dead. The merged image in the stained control group showed blue fluorescence, whereas the merged image in the PDT + hyperthermia group showed intense purple fluorescence, indicating maximum cell death. Normal nuclear morphology was seen in the control group, while condensed and fragmented chromatin were seen in the other treatment groups (PDT + hyperthermia group to the greatest extent) (Figure 9).

The cellular morphology exhibited characteristics consistent with clear cell death, as no viable cells-stained purple were detected. This suggests that hyperthermia treatment in combination with photodynamic therapy (PDT) could be an effective approach (Figure 1). In a similar study, Prasad *et al.* utilized these stains to examine nuclear morphology in HeLa cells 33. Therefore, the multimodal therapeutic approach involving hyperthermia combined with PDT presents a promising strategy for cancer treatment. Furthermore, the system's efficacy can be enhanced by incorporating anticancer drugs into the developed system, leading to potential synergistic effects.

### 3.6. In vivo anti-tumor activity of M-CQDs with MHT and PDT

**Figure 10:**
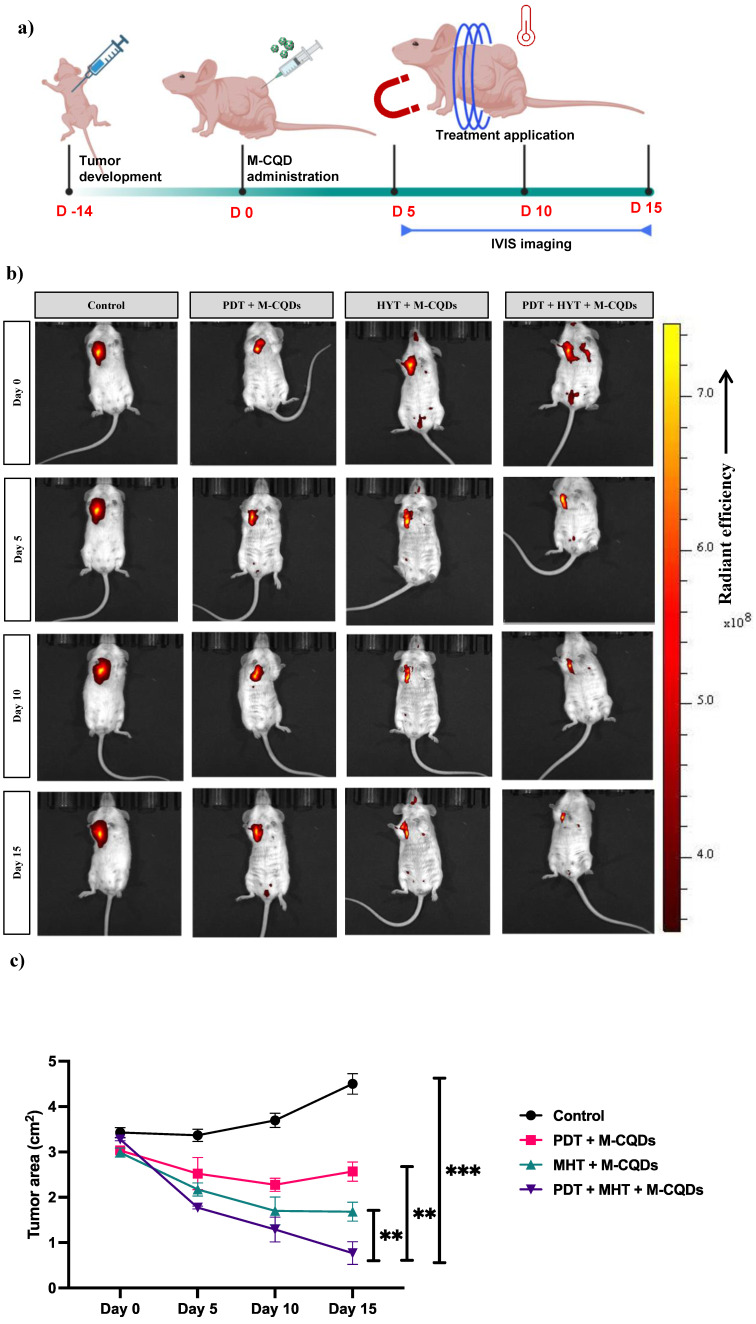
In vivo anti-tumor activity of M-CQDs with MHT and PDT. (a) Animal study design, (b) Representative IVIS imaging of the animals with different treatment application, (c) Tumor area reduction after the different treatments. All the data expressed as mean ± SEM and analysed for statistical significance using one way ANOVA with Tukey post-test Comparison Test, n=3, * vs Control. **p<0.01, ***p< 0.001.

The anti-cancer effect of the M-CQDs combined with MHT and PDT has been evaluated using a mice xenograft model, as shown in the study design (Figure 10 (a)). Further, we have also tracked the 2-DG (2-Deoxy-D-glucose, PerkinElmer) probe using IVIS imaging to investigate the site-specific anti-tumor activity. As depicted in the Figure 10(b), except for the control group in all other groups M-CQDs localized in the tumor region specifically and exhibits the PDT effect. Nevertheless, it has been observed that after day 5, all the groups administered with M-CQDs, started reducing the tumor area. On the similar trends, after day 15, combined MHT and PDT group exhibits highest tumor area reduction compared to only MHT and only PDT application. Taken together these results conclusively proved the efficient site-specific anti-tumor activity of dual-acting M-CQDs.

## 4. Conclusions

In recent times, combination of MHT with PDT has been explored widely as a promising anti-cancer therapy. However, majority of current systems involved toxic photosensitizer, uncontrolled hyperthermia and non-specificity. Herein, in the current study we have developed a dual acting M-CQDs by combining Fe-magnetic nanoparticles combined with amine-carbon quantum dots through the hydrothermal and co-precipitation methods. Next, from the spectroscopic analysis we have confirmed that M-CQDs, exhibit a maximum absorption wavelength (λ_max_) at 288 nm and peak fluorescence intensity observed at 340 nm excitation and 420 nm emission. Further, hyperthermia experiments revelled that both pristine MNPs and M-CQDs could raise the temperature above 42 °C at various frequencies (260, 519, 747, and 995 kHz), with the lowest frequency (i.e., 260 kHz) exhibiting the highest specific absorption rate (SAR) values of 118.11 W/g for MNPs and 95.04 W/g for M-CQDs. Nevertheless, from the cell viability assays we have demonstrated the biosafety of CQDs, MNPs, and M-CQDs even for a longer time-period. Next, the production of ROS induced by A-CQDs upon laser exposure in PDT was assessed using the NBT reduction assay, showing ROS generation at concentrations of 1 mg/ml and 1.5 mg/mL, while M-CQDs exhibited a hindered ROS generation, possibly attributed to interactions between A-CQDs and MNPs. Furthermore, the combination of hyperthermia and PDT treatments resulted in a remarkable cell death rate exceeding 95% as determined by Hoechst and PI staining. At last, using a mice xenograft, we have convincingly proven the localized M-CQDs combined with MHT and PDT effects exhibit the highest anti-tumorigenicity. Conclusively, these findings underscore the potential of multimodal therapies for enhanced cancer treatment efficacy.

## Figures and Tables

**Figure 6:**
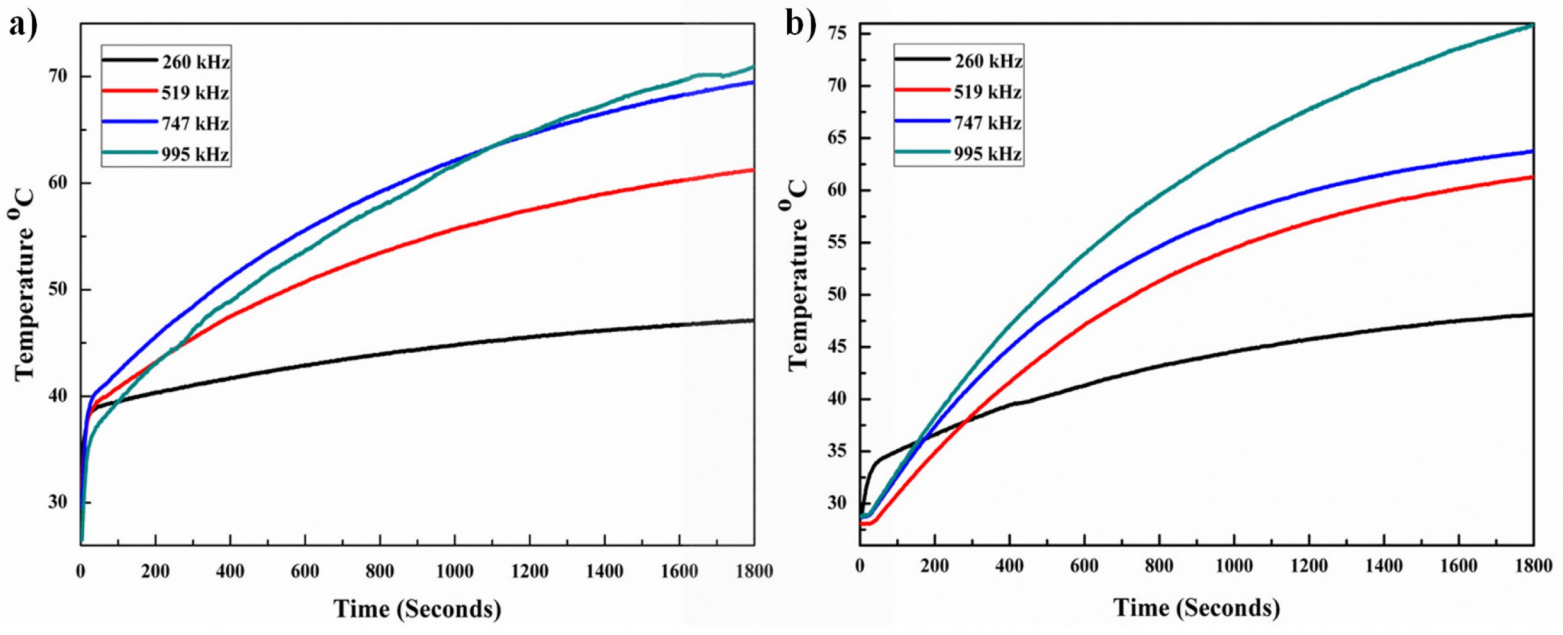
Heating profiles under alternating magnetic field of (a) MNPs and (b) M-CQDs. At low frequency the magnetic fluid shows higher SAR and therapeutic heating for longer time.

**Table 1:** SAR values of MNPs and M-CQDs

Sl. No.	Sample	Frequency (kHz)	Magnetic field (mT)	Time (minutes)		SAR value (W/g)
1	MNPs	995.25	20		30	33.88 ± 1.26
2	MNPs	747.00	20		30	37.91 ± 3.02
3	MNPs	519.10	20		30	50.44 ± 4.38
4	MNPs	260.84	20		30	118.11 ± 3.51
5	M-CQDs	995.25	20		30	32.22 ± 2.75
6	M-CQDs	747.00	20		30	34.91 ± 1.83
7	M-CQDs	519.10	20		30	45.36 ± 2.42
8	M-CQDs	260.84	20		30	95.04 ± 3.16
						
